# Cortical Thickness and Natural Scene Recognition in the Child’s Brain

**DOI:** 10.3390/brainsci10060329

**Published:** 2020-05-28

**Authors:** François Orliac, Grégoire Borst, Grégory Simon, Katell Mevel, Julie Vidal, Sonia Dollfus, Olivier Houdé, Carole Peyrin, Nicolas Poirel

**Affiliations:** 1 Université de Paris, LaPsyDÉ, CNRS, F-75005 Paris, France; orliac@cyceron.fr (F.O.); gregoire.borst@parisdescartes.fr (G.B.); julie.vidal@parisdescartes.fr (J.V.); olivier.houde@parisdescartes.fr (O.H.); 2 GIP Cyceron, 14000 Caen, France; gregory.simon@unicaen.fr (G.S.); katellmevel@hotmail.com (K.M.); dollfus@cyceron.fr (S.D.); 3 Institut Universitaire de France (IUF), 75005 Paris, France; 4 ISTS, UMR 6301, CNRS, CEA, 14000 Caen, France; 5 CHU de Caen, Service de Psychiatrie, Centre Esquirol, 14000 Caen, France; 6 Université Grenoble Alpes, Université Savoie Mont Blanc, CNRS, LPNC, 38000 Grenoble, France; carole.peyrin@univ-grenoble-alpes.fr

**Keywords:** cortical thickness, MRI, children, natural scenes, spatial frequency, vision

## Abstract

Visual scenes are processed in terms of spatial frequencies. Low spatial frequencies (LSF) carry coarse information, whereas high spatial frequencies (HSF) subsequently carry information about fine details. The present magnetic resonance imaging study investigated how cortical thickness covaried with LSF/HSF processing abilities in ten-year-old children and adults. Participants indicated whether natural scenes that were filtered in either LSF or HSF represented outdoor or indoor scenes, while reaction times (RTs) and accuracy measures were recorded. In adults, faster RTs for LSF and HSF images were consistently associated with a thicker cortex (parahippocampal cortex, middle frontal gyrus, and precentral and insula regions for LSF; parahippocampal cortex and fronto-marginal and supramarginal gyri for HSF). On the other hand, in children, faster RTs for HSF were associated with a thicker cortex (posterior cingulate, supramarginal and calcarine cortical regions), whereas faster RTs for LSF were associated with a thinner cortex (subcallosal and insula regions). Increased cortical thickness in adults and children could correspond to an expansion mechanism linked to visual scene processing efficiency. In contrast, lower cortical thickness associated with LSF efficiency in children could correspond to a pruning mechanism reflecting an ongoing maturational process, in agreement with the view that LSF efficiency continues to be refined during childhood. This differing pattern between children and adults appeared to be particularly significant in anterior regions of the brain, in line with the proposed existence of a postero-anterior gradient of brain development. Taken together, our results highlight the dynamic brain processes that allow children and adults to perceive a visual natural scene in a coherent way.

## 1. Introduction

**Figure 1:**
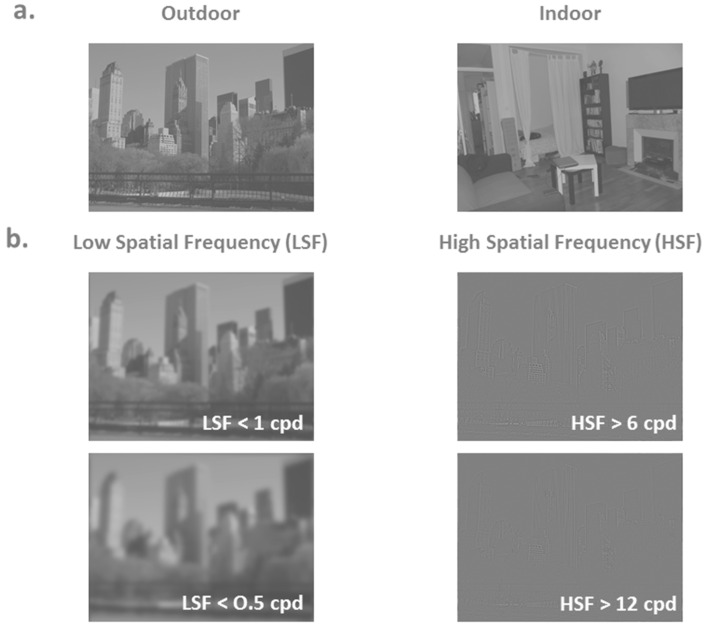
(**a**) Examples of nonfiltered scenes belonging to two different categories (outdoors and indoors) (**b**) Example of low-spatial frequency scenes (LSF) below 0.5 and 1 cycles per degree (cpd) and high-spatial frequency scenes (HSF) above 6 and 12 cpd. It should be noted that the perception of spatial frequencies may be affected by size reduction of the scenes for illustrative purposes.

Recent models of visual perception suggest that scene recognition is processed in terms of spatial frequencies [1,2]. In adults, visual analysis begins with the parallel extraction of different attributes at different spatial frequencies: low spatial frequencies (LSF) provide coarse information about the visual scene, and high spatial frequencies (HSF) provide the details (see Figure 1). There is considerable behavioral evidence suggesting that the rapid processing of LSF would permit initial scene recognition prior to the detailed analysis of fine information in HSF [3,4,5]. In fact, LSF (conveyed by fast magnocellular pathways) could rapidly activate higher-order cerebral areas (e.g., parietal and frontal cortices) and activate plausible semantic interpretations about the stimulus based on coarse information. The orbitofrontal cortex might be predominantly involved in the generation of predictions about the visual stimulus [6,7,8]. Predictions could then be projected to lower order cerebral areas (e.g., occipito-temporal cortex) via top-down connections to guide the subsequent processing of HSF (conveyed more slowly by parvocellular pathways).

At the occipito-temporal level, we now have more knowledge of the regions that are selectively involved in the perception of scenes (compared to those involved in the perception of other stimuli such as faces or objects). Three key brain regions in particular emerge from the literature [9,10,11,12]: the retrosplenial cortex (RSC), the parahippocampal place area (PPA), and the occipital place area (OPA). Critically, scene-selective regions are sensitive to the spatial frequency content of visual scenes [12,13]: the OPA seems selective to HSF and the RSC seems only sensitive to high contrast differences, whereas the PPA responds to an interaction between spatial frequency and contrast in scenes. This functional architecture of the human visual system allows particularly fast predictions about context and promotes rapid access to semantic information during the analysis of visual scenes [14]. In particular, it has been shown that visual processing, such as the recognition of familiar objects and scenes, can be achieved in under 150 ms in adults, regardless of the type of stimulus (biological or manufactured) presented to the participants [15]. As mentioned by Grill-Spector et al. [16], “*As soon as you know it is there, you know what it is*”.

To our knowledge, all the studies that have examined the neural bases of visual scene processing—and, more specifically, from the spatial frequency perspective—were based on a functional approach (i.e., functional MRI and neurophysiology; see Kauffmann et al. [2] for a review). No study has addressed this topic from an anatomical perspective. An anatomical approach could provide new and complementary data concerning visual scene processing and may highlight how these processes evolve between childhood and adulthood with a more dynamic, maturational point of view. This neurodevelopmental perspective appears important in light of previous work reporting that many aspects of visual perception, such as sensitivity to spatial frequencies [17] and perceptual organization [18], further develop after early infancy to become mature and fully efficient during late childhood. Using Navon’s hierarchical stimuli (i.e., large forms composed of local elements [19]), it was shown that the development of children’s visual perceptual processing progressively evolved from a local/HSF preference at four years of age to a more adult-like global/LSF preference around nine years of age [20]. These results suggest that, in late childhood, visual processing shifts from an HSF preference to an LSF preference, an evolution that continues to be refined during adolescence [21], which is consistent with the LSF precedence for scene categorization that has been observed in adults [5,12]. This point raises a few questions, namely, whether the neural regions and networks subtending visual scene processing during childhood are the same as those that have been highlighted in adults. If so, it seems necessary to also investigate the dynamic pattern of the underlying maturational brain processes associated with LSF and HSF processing between children and adults.

In this context, using a sulcogyral parcellation method that provides a measure of the thickness of each brain surface according to the Destrieux atlas [22], the present MRI study aims to investigate, for the first time, the relationship between cortical thickness and behavioral performance regarding LSF/HSF perception in adults and 10-year-old children.

## 2. Methods

### 2.1. Participants

Fourteen healthy children (10.3 ± 0.6 years, five boys, all right-handed) and seventeen healthy adults (30 ± 4.7 years, 10 men, all right-handed) from Caen (France) participated in this study. Information regarding race/ethnicity or nationality were not collected in agreement with national legislation. The participants had no history of major medical condition or neurological disease and no cerebral abnormalities, as assessed by T1-weighted MRI. The local ethics committee approved the study (project identification code: 12-CHUG-02, date of approval: 12 September, 2012, name of the ethics committee: Comité de protection des personnes Sud-Est V, France). Written consent was obtained for all participants. For children specifically, consent was obtained from both parents and children after detailed discussion and explanations.

### 2.2. Behavioral Data

Participants were presented with 80 stimuli filtered either in LSF or in HSF (Figure 1). Stimuli were originally created for a previous study [23]. They consisted of 20 black and white photographs (8-bit greyscale, 1042 × 768 pixels) of scenes classified into two distinct categories (10 indoor scenes and 10 outdoor scenes) with a visual angle of 24 × 18 degrees. Outdoor scenes were views of houses or buildings with sky at the top and outdoor-relevant objects (e.g., car, tree). Indoor scenes were kitchens, offices and living rooms with indoor-relevant objects (e.g., table, sofa, chair). Scenes were displayed in their original version and in their mirrored version (i.e., left and right were reversed) to avoid any effect of the visual asymmetry of these large scene images. Exemplars from the two categories (outdoor and indoor) were chosen to have similar dominant orientations in the amplitude spectrum to avoid categorization based on this type of visual cue. We ensured that all the chosen scenes were equivalent in terms of amplitude spectra and visual cluttering (see Ramanoël et al. [23] for more methodological considerations).

Stimuli were elaborated using the image processing toolbox on MATLAB (MathWorks Inc., Sherborn, MA, USA). Each scene was filtered with two low-pass filters, with cutoff frequencies corresponding to 0.5 and 1 cycles per degree (cpd; i.e., 12 and 24 cycles per image) and two high-pass filters with cutoff frequencies corresponding to 6 and 12 cpd (i.e., 144 and 293 cycles per image). The resulting images were then normalized to obtain a mean luminance equal to 128 on a greyscale ranging from 0 to 256 and a standard deviation of 1, i.e., 25.5 on a greyscale; root mean square (RMS) contrast [24]. This resulted in four versions of each scene (two LSF and two HSF, see Figure 1). Stimuli were displayed using E-Prime software (E-Prime Psychology Software Tools Inc., Pittsburgh, PA, USA). We used a backward mask, built with 1/f white noise, to prevent retinal persistence of the scene.

Each participant performed a total of eight blocks of trials (LSF visual scenes and HSF visual scene conditions). Each block lasted 36 s and included 10 scenes (five indoors and five outdoors). Each scene was presented once in each spatial frequency condition to minimize repetition effects. The order of images was randomized within blocks. Each stimulus was displayed for 100 ms, followed by the white noise mask for 30 ms and a fixation dot in the center of the screen. The interval between the onsets of two successive stimuli was 3600 ms. Participants had to give a categorical answer on the scenes (“indoors” or “outdoors”) by pressing the corresponding key with the forefinger and the middle finger of their dominant hand. They were instructed to fixate on the center of the screen (fixation dot) during the entire experiment and to respond as quickly and as accurately as possible by pressing one of two response buttons. Half of the participants had to answer “indoors” with their forefinger and “outdoors” with their middle finger, while the other half had to answer “indoors” with their middle finger and “outdoors” with their forefinger. The accuracy rate (AR, %) of the responses and reaction times (RTs, ms) were recorded.

### 2.3. Cortical Thickness

Anatomical images were acquired on a 3T MRI scanner (T1-weighted, FOV: 256 mm; slice thickness: 1 mm; 180 slices; matrix size 256 × 256 voxels; TR/TE: 20/4.6; 9 min 41 s duration). The mean cortical thickness of 74 brain regions per hemisphere was estimated for each participant using the Freesurfer 5.1 analysis suite with the Destrieux atlas [22]. For processing, we used optimized intensity nonuniformity correction for 3 Tesla MRI scanners [25] and a process that included visual inspections and the manual correction of topological defects.

## 3. Results

### 3.1. Behavioral Data

RTs and ARs were analyzed with a 2 (age group: children or adults) × 2 (frequency: LSF or HSF) analysis of variance. Adults had faster RTs than children (606 ± 65 ms for adults vs. 977 ± 184 ms for children), F(1,29) = 64.76, *p* < 0.001, η^2^_p_ = 0.69. RTs were faster during the LSF condition (760 ± 118 ms) than the HSF condition (786 ± 154 ms), F(1,29) = 2.92, *p* = 0.098, η^2^_p_ = 0.09, and there was no interaction between the two experimental factors, F(1,29) < 1. Likewise, adults were more accurate than children (96.68% ± 4.71% vs. 92.06% ± 8.20%), F(1,29) = 6.65, *p* = 0.02, η^2^_p_ = 0.19. There was a trend toward more accurate responses during the LSF condition (95.83% ± 3.88% vs. 93.36% ± 8.36%), F(1,29) = 2.68, *p* = 0.11, η^2^_p_ = 0.08, and there was no interaction between the two experimental factors, F(1,29) < 1.

### 3.2. Cortical Thickness

**Figure 2:**
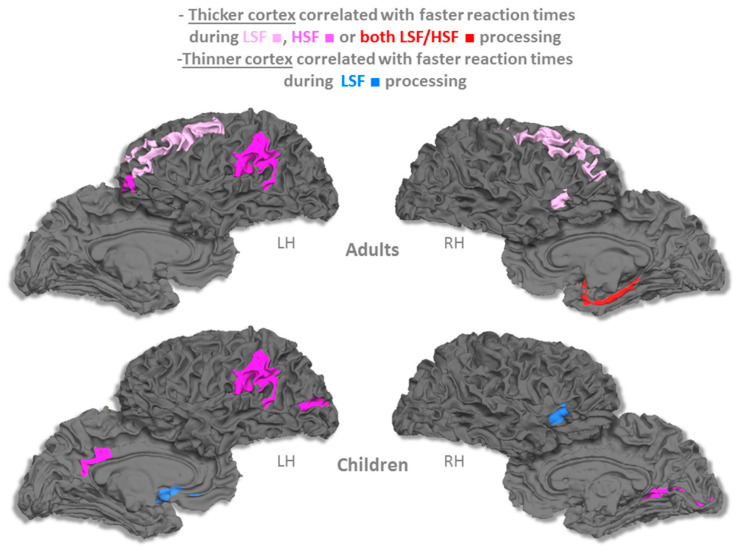
Correlation between the mean cortical thickness values and reaction times in both age groups. HSF: high spatial frequencies; LH: left hemisphere; LSF: low spatial frequencies; and RH: right hemisphere.

For each age group, Pearson’s correlation analyses were carried out for each brain region to investigate the relationship between the mean cortical thickness and LSF/HSF RTs. Cortical thickness and RTs were standardized (*z*-scores) for each age group. The results were reported as statistically significant at *p* < 0.05 (Table 1). In adults, faster RTs were consistently associated with a thicker cortical region (Figure 2). Regardless of spatial frequency, a correlation was found between faster RTs and cortical thickness in the right parahippocampal region. Faster RTs for LSF scenes were also associated with increased cortical thickness in the bilateral middle frontal gyrus, right precentral gyrus and right insula. Faster RTs for HSF scenes were associated with increased cortical thickness in the left fronto-marginal and supramarginal gyri. In sharp contrast, in children, faster RTs during HSF and LSF processing were associated with both thicker and thinner cortical regions, respectively. Faster RTs during HSF processing were associated with increased cortical thickness in posterior areas, i.e., the left posterior cingulate, left supramarginal gyrus, left lunate sulcus and right calcarine sulcus. Faster RTs for LSF scenes were associated with decreased cortical thickness in anterior areas, i.e., the left subcallosal region and right anterior insula.

**Figure 3:**
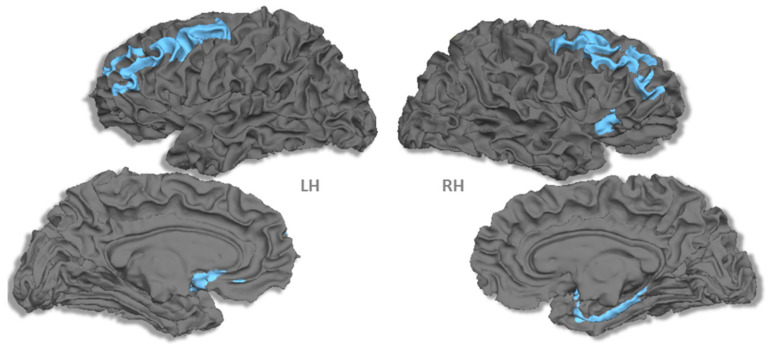
Correlation between the mean cortical thickness values and reaction times that differed between children and adults during LSF processing. LH: left hemisphere; and RH: right hemisphere.

To test for a difference in the cortical thickness × RTs relationship between the two groups (children vs. adults), we carried out an analysis of covariance for each region in which such a correlation between faster RTs and cortical thickness was found, regardless of the age group. We examined the interaction term of the model, in other words, to what extent the regression slopes differed between the two age groups. The results were statistically significant at *p* < 0.05 (Figure 3 and Table 2). During LSF scene processing, the relationships between cortical thickness and RTs were different between the two groups in the bilateral middle frontal gyrus, left subcallosal region, right anterior insula and right parahippocampal region. In these regions, faster RTs were consistently associated with a thicker cortex in adults and with a thinner cortex in children. Concerning HSF, there was no significant effect of age on the cortical thickness × RT relationship.

## 4. Discussion

We investigated, for the first time, the correlation between cortical thickness and the ability to address low (LSF) and high (HSF) spatial frequencies during natural visual scene recognition in adults and children. In adults, faster processing of both LSF and HSF images during visual scene recognition was consistently associated with thicker cortical regions. In sharp contrast, children exhibited a different pattern of results: both increased and decreased cortical thicknesses were found. Interestingly, a specific set of brain regions exhibited a different thickness/RT relationship in children than in adults during LSF processing, characterized by a systematically thicker cortex in adults and a thinner cortex in children. This set of brain regions included (1) the right insula and left subcallosal gyrus, (2) the bilateral middle frontal and right precentral gyrus and (3) the right parahippocampal cortex.

The insula and subcallosal gyrus are part of the orbitofrontal cortex. It has been shown that the orbitofrontal cortex is strongly activated during LSF processing, corresponding to the initial visual information transmitted by the magnocellular visual pathway [26]. The orbitofrontal cortex is also known to play a critical role in facilitating the recognition of visual inputs by sending predictive feedback based on the rapid processing of LSF to sensory cortices [7,8,26]. In the literature, functional segregation is proposed between medial regions (encompassing the anterior cingulate cortex and subcallosal area) and lateral regions (encompassing the inferior frontal cortex and anterior insula). Lateral regions are thought to be engaged in ambiguous stimuli processing [6] and visual predictions [27]. Medial regions of the orbitofrontal cortex are proposed to be more directly related to associative [28] and contextual visual processing [27]. These medial regions, together with the parahippocampal and retrosplenial cortex, are parts of a broader “contextual network” [29] that allows participants to extract the general context of the scene thanks to the information rapidly conveyed by LSF.

Bilateral middle frontal and right precentral gyrus regions encompass frontal eye fields (FEF) and executive regions. FEFs are classically known to be involved in oculomotor control [30,31]. Moreover, Peyrin et al. [32] demonstrated in a combined ERP–fMRI study that FEFs were more highly activated when LSF information was presented before HSF information (i.e., coarse-to-fine vs. fine-to-coarse paradigm). In the same way that the orbitofrontal cortex sends semantic contextual information to lower order cortices, these authors argue that FEFs provide spatial information to sensory cortices to guide HSF analysis and help select the appropriate details for the recognition and categorization of the scene. Executive middle frontal regions are known to be involved in cognitive control during visual perception, particularly for shunting visual information [33] and maintaining a mental image of the environment [34] in the short-term working memory [35].

Finally, we found cortical thickness variations in the parahippocampal cortex, which is located at the junction between brain regions described as essential to memory formation (the hippocampus) and high-level visual processing (the fusiform cortex). More specifically, a subregion of the parahippocampal cortex, the PPA, has been shown to be a scene-selective region [11]. This region is known to be involved in the representation of visual scenes [10] and contextual associations (see [2] for a review). In line with the present finding, Musel et al. [36] reported that the processing of LSF before HSF may constitute the predominant strategy for scene categorization in this region.

Apart from this set of regions that exhibit a different pattern in children and adults, the present results also highlighted the involvement of the lateral occipital cortex and posterodorsal cingulate cortex (i.e., RSC) for HSF processing in children, with a thicker cortex being linked to faster RTs. The anatomical region labeled “middle occipital sulcus and lunatus” in the Destrieux atlas overlaps with the OPA [37,38], which is known to be selectively involved in scene perception [9] and, more precisely, in HSF processing [12,39]. In the same vein, RSC activity has been linked to visuo-spatial memory processes [40]. Finally, the present results also demonstrated the involvement of two brain regions, the left supramarginal gyrus (in children and adults) and the right calcarine (in children only), in HSF processing, with a thicker cortex being linked to faster RTs. These results could correspond to the automatic allocation of attention [41] and the fine-tuning of the right primary visual cortex in children [42], respectively.

From a developmental point of view, and beyond the description of the aforementioned brain network involved in natural scene recognition, the present study highlights two different maturational brain mechanisms in children and adults. On one hand, in adults, visual expertise during visual scene recognition (involving both HSF and LSF processes) was consistently associated with increased cortical thickness in associated regions. In adults, better abilities or improvement in abilities following training have been associated with thicker cortical regions [43,44]. Several mechanisms can account for this macroscopic expansion: synaptic changes (axon sprouting and dendritic branching), glial activation and proliferation, and in some brain regions such as hippocampi, neurogenesis [45]. On the other hand, children exhibited a different pattern of results: cortical regions were found to be either thicker or thinner. This suggests that, as for adults, a thicker cortex may correspond to an expansion mechanism, specifically for HSF processing, a process that seems to become efficient by 10 years of age based on evidence using compound stimuli [20]. In contrast, in children, faster RTs during LSF processing were associated with thinner cortical regions, opposite to what was found for HSF. In healthy children, cortical thinning is now seen as a reliable marker of maturation. It may represent two concurrent processes: synaptic pruning (leading to a reduction in the number of glial cells) and myelination of intra-cortical fibers [46]. The thinner cortex associated with LSF efficiency in children may reflect an ongoing maturational process, which is consistent with the fact that LSF efficiency continues to be refined during childhood and adolescence [47,48]. Importantly, this pattern difference between children and adults is particularly significant in anterior regions of the brain, suggesting that the scene recognition domain follows the well-known postero-anterior maturational gradient of brain development during childhood [48,49]. Along with the maturation of anterior regions, contextual, associative and predictive coding may become more efficient, leading to the faster recognition of complex scenes. Surprisingly, we found few associations between scene recognition and cortical thickness in primary visual regions. However, given that (a) cortical maturation occurs first in sensorimotor areas, followed by association areas, and lastly by higher-ordered cortical areas [46] and that (b) the primary visual cortex is mature at the age of ten [22], which is the mean age in the children group, scene recognition most probably rely on higher order associative regions still not mature at the age of 10.

A limitation of the present study is the small sample size and the use of uncorrected thresholds, which may lead to false positive results. Nevertheless, most of the brain regions highlighted in the present study are already known to be involved in scene perception in adults, and to be sensitive to spatial frequency content of visual scenes [12,13]. The present study provides preliminary evidence into the brain maturational processes involved in scene perception. Our findings need to be refined by findings from longitudinal studies from childhood to early adulthood with larger sample sizes using different stimuli (e.g., Navon figures) including control stimuli from other modalities (e.g., auditory).

## 5. Conclusions

In conclusion, the present results provide new insights into both brain network and brain dynamic processes that allow children and adults to recognize and categorize a visual natural scene.

## Figures and Tables

**Table 1:** Correlation between the mean cortical thickness values and reaction times within the regions of interest according to the Destrieux atlas [22].

	Label	Hemisphere	*p*-Value	Pearson’s ρ
Adults				
LSF				
	G_front_middle	L	0.046	−0.49
	G_front_middle	R	0.048	−0.49
	G_oc-temp_med-Parahip	R	0.024	−0.55
	S_circular_insula_ant	R	0.021	−0.55
	S_precentral-sup-part	R	0.026	−0.54
HSF				
	G_and_S_frontomargin	L	0.046	−0.49
	G_pariet_inf-Supramar	L	0.025	−0.54
	G_oc-temp_med-Parahip	R	0.030	−0.53
				
Children				
LSF				
	G_subcallosal	L	0.031	0.58
	S_circular_insula_ant	R	0.005	0.70
HSF				
	G_cingul-Post-dorsal	L	0.033	−0.57
	G_pariet_inf-Supramar	L	0.018	−0.62
	S_oc_middle_and_Lunatus	L	0.033	−0.57
	S_calcarine	R	0.027	−0.59

All results are statistically significant at *p* < 0.05. G: gyrus; HSF: high spatial frequencies; L: left hemisphere; LSF: low spatial frequencies; R: right hemisphere; and S: sulcus.

**Table 2:** Effect of age group (children vs. adults) on the cortical thickness × RT relationship during LSF processing. Significance values refer to the interaction term of an analysis of covariance between cortical thickness (dependent variable), age group (independent variable) and RTs (covariate), using a “separate lines” model.

Label	Hemisphere	F(1,27)	*p*-Value	η^2^_p_
G_front_middle	L	4.98	0.03	0.16
G_subcallosal	L	7.79	0.01	0.22
G_front_middle	R	4.35	0.047	0.14
G_oc-temp_med-Parahip	R	5.92	0.02	0.18
S_circular_insula_ant	R	17.30	<0.001	0.39

All results are statistically significant at *p* < 0.05. LSF: low spatial frequencies; and RT: reaction time.

## Data Availability

The datasets generated during the current study are available from the corresponding author on reasonable request.

## Abbreviations

HSF	high spatial frequencies
LSF	low spatial frequencies
OPA	occipital place area
PPA	parahippocampal place area
RSC	retrosplenial cortex
RTs	reaction times
